# Recurrence of pancreatic cancer presented as cervical lymphadenopathy

**DOI:** 10.1186/s12957-016-0773-0

**Published:** 2016-01-20

**Authors:** Hiroshi Nagata, Ken Hayashi, Shigetoshi Yamada

**Affiliations:** Department of General Surgery, Kameda Medical Center, 929, Higashi-cho, Kamogawa, Chiba Japan

**Keywords:** Pancreatic cancer, Metastasis, Multimodal treatment

## Abstract

**Background:**

We report a case of recurrent pancreatic cancer that presented as cervical lymphadenopathy.

**Case presentation:**

A 69-year-old woman with stage IIb pancreatic cancer underwent a curative Whipple’s procedure after neoadjuvant chemoradiation therapy. Despite adjuvant chemotherapy with S-1, postoperative recurrence was diagnosed as left cervical lymphadenopathy 11 months postoperatively. After she underwent chemoradiation therapy to the cervical area followed by systemic chemotherapy with S-1, the lymphadenopathy became unremarkable 17 months postoperatively. S-1 treatment was discontinued 23 months postoperatively at the patient’s request. She has remained free of disease since that time and has achieved an overall duration of survival of 48 months.

**Conclusions:**

To the best of our knowledge, this is the first reported case of middle cervical lymph node metastasis of pancreatic cancer. Although rare, it should be considered as a site of recurrence. This case suggests concurrent radiation therapy can be a good option for patients who cannot tolerate an aggressive regimen.

## Background

Despite advances in surgical techniques and perioperative care, the probability of long-term survival in patients with pancreatic adenocarcinoma remains low because of the high relapse rate. We report a rare case of recurrent pancreatic cancer that presented as left cervical lymphadenopathy. The patient has achieved postoperative survival of 48 months as a result of multimodal treatment.

## Case presentation

**Fig. 1:**
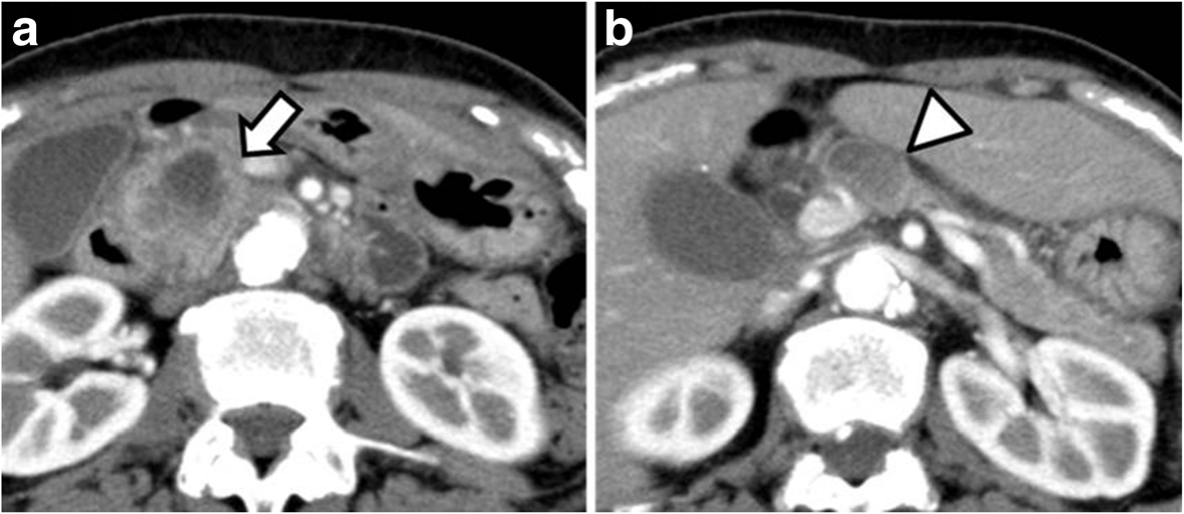
CT showing the primary tumor (**a**; *arrow*) and a swollen lymph node (**b**; *arrowhead*)

A 69-year-old woman presented to our clinic with a chief complaint of epigastric pain. She had a history of squamous cell carcinoma of the mandibular gingiva, which had been completely resected 2 months prior to the visit (T1N0M0, stage 1). Her laboratory data were normal except for elevated amylase (327 U/L; upper normal limit (UNL) <125 IU/L) and DUPAN-2 (16,000 U/mL; UNL <150 U/mL) levels. Contrast-enhanced computed tomography (CT) showed a 45-mm low-attenuating tumor in the head of the pancreas, causing dilatation of the pancreatic duct and common bile duct (Fig. 1a). CT further revealed a 24-mm lymph node along the common hepatic artery (Fig. 1b). No distant metastasis was recognized. An endoscopic ultrasound-guided fine-needle aspiration biopsy of the lymph node revealed a well-differentiated tubular adenocarcinoma. Since tumor invasion to the pancreatic capsule was suspected and the contour of the superior mesenteric vein (SMV) was irregular, the clinical stage was Ph, TS3, T4 (CH+, DU−, S+, RP+, PV+, A−, PL+, OO−), N2M0, cStage IVb (following the Japan Pancreas Society (JPS) classification [1]), or T3N1M0, stage IIb (following the Union for International Cancer Control (UICC) [2]).

**Fig. 2:**
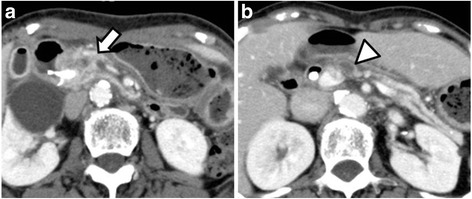
CT images after chemoradiation therapy (**a**, **b**)

The patient received neoadjuvant chemoradiation therapy (CRT: S-1, 100 mg/day × 28 days + radiation therapy (RT) 50.4 Gy/28 Fr) for the borderline resectable pancreatic cancer. After CRT, the DUPAN-2 level decreased to 960 U/mL. The tumor shrunk to a diameter of 18 mm, and the contour between the SMV and the tumor became clear (Fig. 2). The disease stage following CRT was TS1, T3 (CH+, DU−, S−, RP−, PV−, A−, PL−, OO−) N2M0, cStage IVa (JPS), or T1N1M0, stage IIb (UICC). The patient underwent subtotal stomach-preserving pancreaticoduodenectomy with D2 dissection 3 weeks after CRT. Pathological examination revealed that the primary tumor and the lymph node had become necrotic, and no residual viable cancer cells were recognized. The pathological diagnosis was invasive ductal adenocarcinoma, TS1, T1 (CH−, DU−, S−, PV−, A−, PL−, OO−) N0M0, pStage I (JPS), or T1N0M0, stage I (UICC), and R0 resection was confirmed. Gemcitabine was discontinued after two courses because of grade 3 neutropenia, and the patient therefore received S-1 (80 mg/day) for 6 months as adjuvant chemotherapy.

**Fig. 3:**
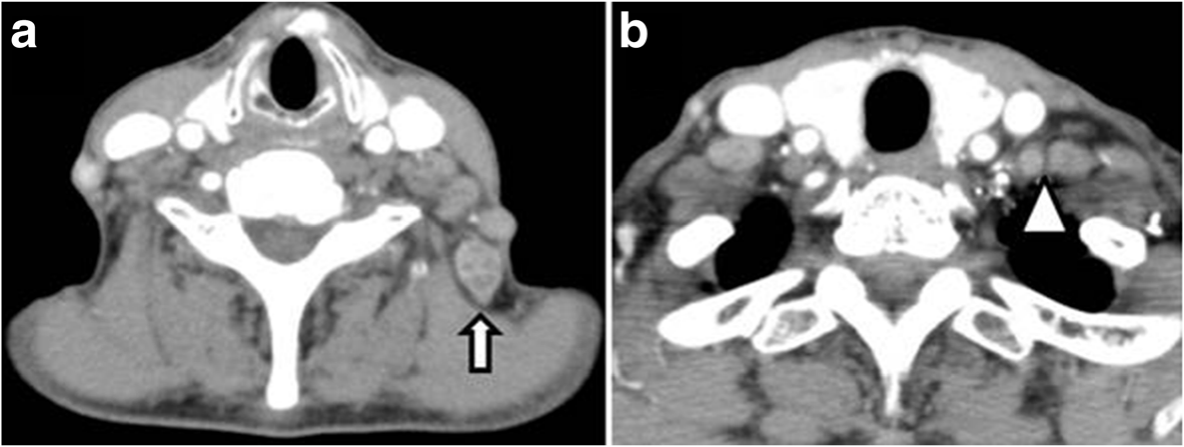
Adenopathy of a left accessory nerve lymph node (**a**; *arrow*) and middle deep cervical lymph nodes (**b**; *arrowhead*)

**Fig. 4:**
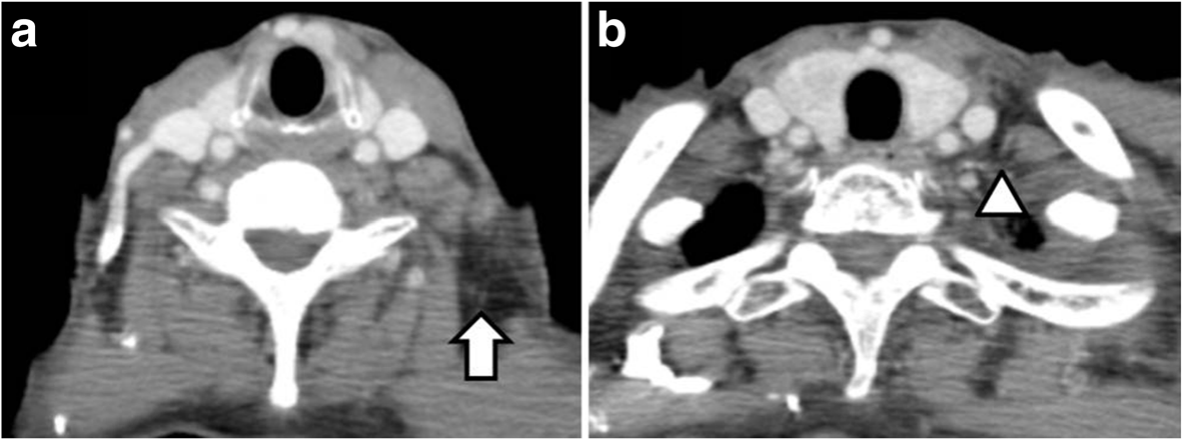
CT after chemoradiation therapy showing complete response to the treatment (**a**, **b**)

Four months after the discontinuation of S-1 (11 months postoperatively), CT revealed adenopathy of a left accessory nerve lymph node and deep cervical lymph nodes. Further, positron-emission tomography (PET)-CT revealed an abnormal accumulation at these lymph nodes (Fig. 3). The DUPAN-2 level was elevated to 4460 U/mL. Aspiration biopsy of the lymph nodes revealed adenocarcinoma with an immunohistochemical pattern that was compatible with the preceding pancreatic cancer; thus, postoperative recurrence was confirmed. S-1 therapy (80 mg/day) was restarted and concurrent RT (50.4 Gy/28 Fr) to the cervical area was adopted for local control. The lymphadenopathy became unremarkable on a CT scan obtained 17 months postoperatively (Fig. 4), and the DUPAN-2 level decreased to normal. S-1 treatment was discontinued 23 months postoperatively at the patient’s request. She has been disease free since that time and has achieved an overall survival of 48 months.

## Discussion

We present a rare case of recurrent pancreatic cancer, in which recurrence presented as left cervical lymphadenopathy, and the patient achieved an overall survival of 48 months. Considering that the median postoperative survival time for stage IVb (JPS) pancreatic cancer is only 11.7 months [3], this case is remarkable.

Postoperative recurrences of pancreatic cancer can be categorized as either local recurrence or distant metastasis. Common sites of the latter include the liver, peritoneum, para-aortic lymph nodes, and lung [4]. To date, about ten cases of cervical lymph node metastasis have been reported [5–7] but in all of these cases, metastasis was noted in the supraclavicular lymph nodes. To the best of our knowledge, this is the first reported case of middle cervical lymph node metastasis of pancreatic cancer. Generally, pancreatic cancer is known to metastasize rapidly to the lymphatic system. The exact mechanism of the spread has not been elucidated, but it is probably caused by permeation, embolization, and retrograde spread due to lymphatic obstruction in the pancreas [7]. The route of metastasis to the middle cervical lymph node is not clear, but provided that the cancer cells spread through the mediastinal lymph nodes to the supraclavicular lymph node, they can cause middle lymph node metastasis along the way. Cervical lymph node metastases can be overlooked because CT scans performed during normal postoperative follow-up often omit the cervical area, and PET-CT scans are not routinely performed.

In our case, the most likely differential diagnosis of cervical lymphadenopathy should be metastasis of mandibular gingival squamous cell carcinoma because as much as 7 % of such cases can involve postoperative lymph node metastasis [8], even in cases of T1 stage disease. However, this possibility was ruled out by examination of the lymph node biopsy specimen, which revealed adenocarcinoma. Another possibility was metastasis of occult adenocarcinoma. The alternative was also unlikely in our case because esophagogastroduodenoscopy and CT ruled out the presence of any other primary lesion, and the immunohistochemical profile of the lymph node was consistent with the primary pancreatic cancer.

While the Japanese clinical guidelines for pancreatic cancer lump recurrent disease with metastatic or unresectable disease [9], the National Comprehensive Cancer Network guidelines propose specific treatment strategies for postoperative recurrence [10]. For distant metastasis that occurs less than 6 months after the completion of primary therapy, the guidelines recommend enrollment of the patient in a clinical trial and list alternative systemic chemotherapy and best supportive care as options. In the present case, however, re-administration of S-1 was the only choice because our patient could not tolerate gemcitabine.

S-1 has been the key drug for treating pancreatic cancer since the JASPAC-01 study revealed its superiority to gemcitabine in the adjuvant chemotherapy setting in Japan [11]. We chose to add concurrent radiation for local control in order to intensify the antitumor activity. In principle, RT for recurrent disease is limited to local recurrence and palliative care, while distant metastasis should be treated as a systemic disease. In practice, however, aggressive chemotherapy is often a difficult choice for fragile patients with recurrent pancreatic cancer. We expect that the effects of S-1 contributed greatly to the good outcome in our patient; yet, at the same time, this case implies that concurrent RT is a potent option for localized distant metastasis.

## Conclusions

We have described a rare case in which recurrent pancreatic cancer presented as left cervical lymphadenopathy in a patient who achieved an overall survival of 48 months.

### Consent

Written informed consent was obtained from the patient for publication of this case report and any accompanying images. A copy of the written consent is available for review by the Editor-in-Chief of this journal.

## Abbreviation

CRT: chemoradiation therapy
CT: computed tomography
JPS: Japan Pancreas Society
SMV: superior mesenteric vein
UICC: Union for International Cancer Control
